# The Specialized Proresolving Mediator Maresin 1 Alleviated Neuropathic Pain Exacerbated by High‐Fat Diet via Regulating Neuroinflammation

**DOI:** 10.1155/prm/1157250

**Published:** 2026-08-12

**Authors:** Li Zhang, Ran Hu, Kexin Qin, Xue Wang, Chen Wu, Zichen Liu, Xiangqi Shao, Jianru Sun, Yang Liu, Saisong Xiao, Huilong Ren, Fan Liu, Guokai Liu

**Affiliations:** ^1^ Department of Anesthesiology and Operating Room, Xiyuan Hospital, China Academy of Chinese Medical Sciences, Beijing 100091, China, cacms.ac.cn; ^2^ Department of Nephrology, the First Affiliated Hospital of Anhui University of Chinese Medicine, Hefei 230031, China, ahtcm.edu.cn; ^3^ Dongzhimen Hospital, Beijing University of Chinese Medicine, Beijing 100700, China, bucm.edu.cn; ^4^ Department of Human Anatomy, Histology and Embryology, Institute of Basic Medical Sciences & School of Basic Medicine, Chinese Academy of Medical Sciences & Peking Union Medical College, Beijing 100005, China, cams.ac.cn; ^5^ Beijing Friendship Hospital, Capital Medical University, Beijing 100050, China, ccmu.edu.cn; ^6^ The First Clinical Medicine Faculty, China Medical University, Shenyang 110122, China, cmu.edu.tw

**Keywords:** chronic pain, dorsal root ganglion, growth-associated protein 43, macrophage subtypes, neuroregeneration

## Abstract

**Background:**

Clinical observations have suggested an association between a high‐fat diet (HFD) and neuropathic pain (NP). Studies also revealed that maresin 1 (MaR1) can regulate macrophage phagocytosis, modulate tissue repair and inflammatory resolution, and influence the progression of NP. However, the specific regulatory mechanisms of MaR1 in HFD‐induced NP remain unclear.

**Objective:**

The aim of this study is to explore the effect of MaR1 on neuroinflammation in mice with NP induced by an HFD.

**Methods:**

A tibial nerve crush model with a HFD was used to establish HFD‐induced chronic pain. The paw withdrawal threshold (PWT), which reflects mechanical allodynia, was measured to evaluate changes in pain‐related behavior. Immunofluorescence was used to detect the relative number of macrophages and microglia, and the expression of growth‐associated protein 43 (GAP43), while qPCR was used to measure the levels of proinflammatory cytokines.

**Results:**

HFD consumption exacerbates nerve injury–induced NP formation and upregulates GAP43 expression. Macrophage infiltration and proinflammatory cytokine expression were increased in the DRGs of HFD‐induced nerve injury mice. Intrathecal injection of the specialized proresolving mediator MaR1 alleviated nerve injury–induced chronic pain and decreased GAP43 expression in HFD‐fed mice. Furthermore, MaR1 decreased proinflammatory macrophage migration in the damaged tibial nerves and DRGs of HFD‐induced nerve injury model mice.

**Conclusions:**

The specialized proresolving mediator MaR1 can reduce neuroinflammation and GAP43 expression in the DRG and injured nerve fibers and alleviate NP in HFD‐fed mice.

## 1. Introduction

Neuropathic pain (NP) is mainly caused by nerve damage, inflammation, or other lesions of the nervous system and is often chronic and difficult to treat. Studies suggest that neurogenic obesity exacerbates NP after spinal cord injury (SPI) by inducing metabolic disorders and promoting the secretion of inflammatory factors, including TNF‐α, IL‐1β, IL‐6, monocyte chemoattractant protein‐1 (MCP‐1), and NF‐κB [1, 2]. These inflammatory mediators activate transient receptor potential (TRP) channels (e.g., TRPA1, TRPV1) and sodium channels (e.g., Nav1.7, Nav1.8) in peripheral nociceptive neurons, enhancing neuronal sensitivity and promoting macrophage infiltration, further amplifying neuroinflammation [3, 4].

CX3CR1^+^‐resident macrophages play a crucial role in NP [5, 6]. Their proliferation in the dorsal root ganglia (DRGs) contributes to the persistence of NP by serving as a major source of inflammatory cytokines such as TNF‐α and IL‐6 [7]. Cd68^+^ macrophages can differentiate into proinflammatory M1 type (expressing IL‐1β, TNF‐α, and IL‐6) or anti‐inflammatory M2 type (expressing IL‐10 and TGF‐β). In NP, the Cd68^+^ macrophages tend to differentiate into the M1 type and are closely associated with neuroinflammation [8].

Additionally, microglia in the spinal dorsal horn are activated by colony‐stimulating factor 1 (CSF1) after nerve injury, leading to mechanical allodynia [9]. Activated microglia further produce inflammatory mediators, exacerbating NP. Inhibition of CSF1 signaling has been shown to suppress both peripheral macrophages and microglia, alleviating NP [10].

Uncontrolled neuroinflammation after nerve injury can cause irreversible damage, highlighting the need for early intervention [11]. Maresin 1 (MaR1), a macrophage‐derived proresolving lipid mediator, exerts strong anti‐inflammatory and tissue‐repairing effects. In SCI, intravenous MaR1 reduces proinflammatory macrophages while sparing anti‐inflammatory ones, downregulates key cytokines (CXCL1, CXCL2, CCL3, CCL4, IL‐6, CSF3), and improves motor function [12]. In a sciatic nerve crush model, MaR1 promotes neuronal regeneration via the PI3K–AKT–mTOR pathway and inhibits spinal microglia and astrocyte activation, lowering IL‐1β, IL‐6, and TNF‐α levels to relieve NP [13].

Growth‐associated protein 43 (GAP43), a marker of nerve regenerative capacity, is involved in axonal growth and synaptic remodeling but may also contribute to NP [14]. Studies suggest that nicotine downregulates GAP43 expression and alleviates allodynia in tibial nerve compression models [15], while microsympathectomy reduces GAP43 levels in DRGs and mitigates mechanical allodynia after nerve injury [16]. However, the precise role of GAP43 in NP remains unclear.

Taken together, MaR1 exerts anti‐inflammatory effects by regulating macrophage polarization and suppressing neuroinflammation, thereby alleviating NP. However, its mechanism in obesity‐related NP remains unclear. This study investigates how MaR1 modulates macrophage phenotypic switching and GAP43 expression in obese individuals with nerve injury, providing insight into its potential clinical applications in NP management.

## 2. Materials and Methods

### 2.1. Animals

Specific pathogen‐free (SPF) wild‐type male C57BL/6 mice, aged 7 weeks and weighing 20–25 g, were used in this study. Animals were randomly allocated to each group (*n* = 8 per group). These mice were bred in a pathogen‐free environment with a 12‐h light–dark cycle. Mice on a high‐fat diet (HFD) were fed a semisynthetic high‐fat feed, with 40% of the calories coming from fat, primarily from milk fat. The remaining mice were provided with a standard diet (SD). HFD feeding was initiated 6 weeks prior to surgery and continued throughout the postoperative observation period. All animal procedures performed in this study were approved by the Animal Ethical Committee of Beijing Friendship Hospital, Capital Medical University (20–1004), according to the guidelines of the International Association for the Study of Pain (IASP).

### 2.2. Animal Model of NP

After intraperitoneal injection of sodium pentobarbital for anesthesia, an incision was made along the axis of the femur to expose the biceps femoris muscle under aseptic conditions. The sciatic nerve was then isolated, and the tibial nerve was separated using glass forceps. The tibial nerve was compressed using flat forceps for 60 s. The tibial nerve crush (TNC) injury model induces reversible damage and serves as an effective model for nerve regeneration. The Sham group only incised the skin and muscles and then sutured them without compressing the tibial nerve. On the first day after surgery, MaR1 (Cayman Chemical, USA; 100 ng/10 μL) [17] was intrathecally injected using a micro syringe, with continuous treatment for 3 days.

### 2.3. Behavioral Assessment of Pain

The paw withdrawal threshold (PWT) of eight mice in each group was used to assess mechanical allodynia. The first measurement was taken using Von Frey filaments before surgery, and subsequent measurements were taken every 4 days postsurgery until Day 36. Before each test, every mouse was allowed to acclimate for 1 h in an organic glass box, and bilateral paws were tested three times with 5‐min intervals between each test. The average of the three values of the same side was taken as the PWT. The entire testing process was conducted in a quiet environment at a room temperature ranging from 23°C to 25°C. On postoperative Days 7 and 28, L4–L6 DRGs and the injured tibial nerves were collected from the mice and rapidly frozen at −80°C.

### 2.4. Immunofluorescence Staining and Quantification

After the mice were dissected and fixed, the bilateral L4–L6 DRGs and injured tibial nerves were extracted. The tissues were then placed in 4% paraformaldehyde for 2 h for fixation and dehydrated in a 30% sucrose solution at 4°C. The tissues were embedded in optimal cutting temperature compound (SAKURA, Catalog No. 4583) and then sliced into 12‐μm sections using a cryostat. After the sections were permeabilized and blocked, they were incubated with primary antibodies against IBA1 (1:500, Abcam, Catalog No. ab178846), CX3CR1 (1:200, Abcam, Catalog No. ab308613), or GAP43 (1:200, Abcam, Catalog No. ab75810) overnight at 4°C. Then, the sections were stained with Alexa Fluor 594‐conjugated goat anti‐rabbit secondary antibodies (1:400, Abcam, Catalog No. ab150080) or Alexa Fluor 488–conjugated goat anti‐rabbit secondary antibodies (1:400, Abcam, Catalog No. ab150077) for 1 h. Finally, the sections were washed in PBS and cover‐slipped with VECTASHIELD Mounting Medium with DAPI (Cat: H‐1200, Vector Lab). Images were captured by a microscopic imaging system (Olympus BX61 and FluoView). Fluorescence intensity was quantified using ImageJ software (National Institutes of Health, USA). For each section, 3–5 random nonoverlapping fields of view were selected within the anatomical regions of interest (ROIs), specifically the DRGs and the tibial nerve injury site. Images were converted to 8‐bit grayscale. To ensure consistency, a uniform threshold was applied across all images to eliminate background noise and define positive signals. The mean fluorescence intensity was measured for each ROI, and data were averaged from at least three independent sections per animal.

### 2.5. Quantitative Real‐Time PCR

Total RNA was collected from ipsilateral and contralateral DRGs and damaged tibial nerves with TRIzol reagent (Invitrogen Life Technologies) and reverse‐transcribed using a reaction mixture in accordance with the manufacturer’s instructions. RNA quality and quantity were determined with a NanoDrop spectrophotometer (ND‐1000; NanoDrop Technologies), and RNA integrity was assessed through gel electrophoresis. RNA was reverse‐transcribed using PrimeScript RT Master Mix (RR036A, Takara Bio). Subsequently, the target genes were amplified using TB Green Premix Ex Taq II (RR820A, Takara Bio). Quantitative RT‐PCR (qPCR) was performed on a Bio‐Rad CFX96 instrument. The expression data were normalized to the expression of *GAPDH*, and relative changes in mRNA expression were measured using the comparative threshold cycle (Ct) method and 2^−ΔΔCt^. Each sample was measured in at least three technical replicates, and each group included eight biological replicates. All primers were synthesized by Sangon Biotech (Shanghai) Co., Ltd., and the sequences of the primers used for qPCR are as follows: *GAPDH-*sense (GTCCCTCACCCTCCCAAAAG) and antisense (GCTGCCTCAACACCTCAACCC), *Il1b-*sense (CAACCAACAAGTGATATTCTCCATG) and antisense (GATCCACACTCTCCAGCTGCA), and *Cd68-*sense (TGTCTGATCTTGCTAGGACCG) and antisense (GAGAGTAACGGCCTTTTTGTGA).

### 2.6. Statistical Analysis

All the data are expressed as the means ± SEMs, and the PWTs were log‐transformed to satisfy the assumption of normality. GraphPad Prism 5.0 (San Diego, CA, USA) software was used for all analyses. The normality of data was assessed using the Shapiro–Wilk test prior to analysis. For data that met the normality assumption, behavioral data were analyzed using two‐way repeated‐measures ANOVA, while qPCR and immunofluorescence data were analyzed using one‐way or two‐way ANOVA as appropriate, followed by Bonferroni post hoc correction. *p*  <  0.05 was considered to indicate statistical significance.

## 3. Results

### 3.1. A HFD Exacerbates Nerve Injury–Induced NP and Upregulates GAP43 Expression

**FIGURE 1:**
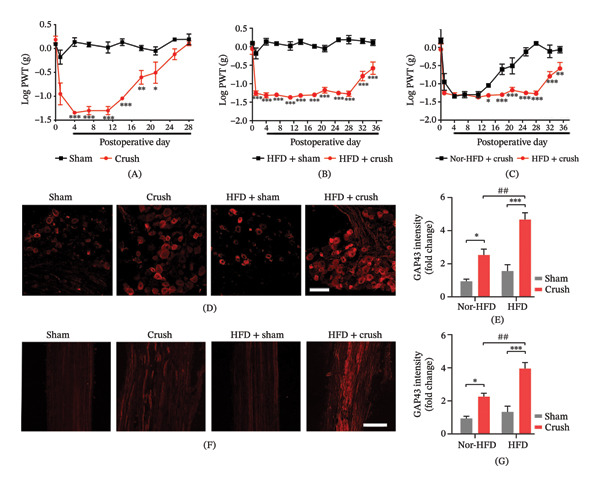
High‐fat diet exacerbates mechanical allodynia and upregulates GAP43 expression in the DRG and tibial nerve induced by crush injury. (A–C) Assessment of mechanical allodynia using PWT. (A) Crush injury induced a rapid reduction in PWT with gradual recovery beginning on POD 14 in the crush group. (B) HFD‐fed mice exhibited prolonged and pronounced hyperalgesia compared to the HFD‐sham group. (C) HFD significantly exacerbated mechanical allodynia and delayed recovery until POD 28 compared to the Nor‐HFD + crush group. Data are presented as mean ± SEM. Two‐way repeated‐measures ANOVA followed by Bonferroni post hoc test was used for statistical analysis. ^∗^ *p* < 0.05, ^∗∗^ *p* < 0.01, ^∗∗∗^ *p* < 0.001 vs. control groups. (D–G) Immunofluorescence images and quantitative analysis of GAP43 expression in the DRG and tibial nerve on POD 28. (D, E) Compared to the sham group, crush injury significantly increased GAP43 expression in DRG neurons, and this effect was exacerbated by HFD. (F, G) GAP43 expression in the tibial nerve was markedly enhanced in the HFD‐crush group compared to the Nor‐HFD + crush group. Data are presented as mean ± SEM. Two‐way repeated‐measures ANOVA followed by Bonferroni post hoc test was used for statistical analysis. Two‐way ANOVA, ^∗^ *p* < 0.05, ^∗∗∗^ *p* < 0.001 vs. sham; ^##^ *p* < 0.01 vs. Nor‐HFD group. *n* = 8 per group. Nor‐HFD: normal (non‐high‐fat diet) group.

GAP43 is a well‐established marker associated with axonal growth and regenerative capacity during nerve repair. Our study revealed that GAP43 levels in the DRG increased significantly after crush injury. To explore the impact of GAP43 on chronic pain after nerve injury caused by a HFD, we used a TNC injury model to induce pathological NP. Compared to the sham group, the crush group exhibited rapid and severe mechanical allodynia, which began to gradually recover on POD14 (Figure 1A). However, in mice fed an HFD, compared to those in the sham group, crush‐induced hyperalgesia was more prolonged and pronounced (Figure 1B). Additionally, the HFD exacerbated mechanical allodynia after crush and delayed the recovery process, with no signs of amelioration appearing until POD28 (Figure 1C).

We further examined GAP43 expression in the DRG and tibial nerve fibers on POD28 by immunofluorescence. After crush injury, GAP43 expression in DRG neurons was significantly greater in the crush group than in the sham group, and this effect was exacerbated by the HFD, leading to a significant increase in the proportion of GAP43‐positive neurons after the crush procedure (Figure 1D,E). Similar changes were also observed in the tibial nerve fibers. GAP43 expression in nerve fibers increased after the crush procedure, and this increase was markedly enhanced by an HFD (Figure 1F,G).

### 3.2. A HFD Increased Macrophage Infiltration and Proinflammatory Cytokine Expression in the Dorsal Root Ganglion of Nerve Injury Model Mice

**FIGURE 2:**
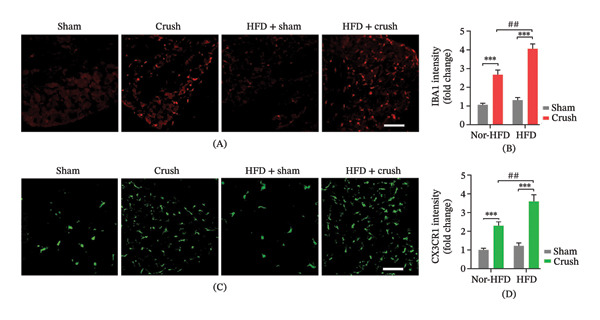
High‐fat diet exacerbates macrophage infiltration in the DRG induced by crush injury. (A–D) Immunofluorescence images and quantitative analysis of macrophage subpopulations in the DRG on POD 28. (A, B) IBA1^+^ macrophages. Crush injury elicited significant macrophage recruitment, which was markedly exacerbated by HFD feeding. (C, D) CX3CR1^+^ macrophages. The proportion of these cells was significantly increased in the HFD + crush group compared to the Nor‐HFD + crush group. Two‐way ANOVA, ^∗∗∗^ *p* < 0.001 vs. Sham; ^##^ *p* < 0.01 vs. Nor‐HFD group. *n* = 8 per group.

Our previous research confirmed that following peripheral nerve injury, macrophages migrate and infiltrate the peripheral ganglia, leading to the onset of neuroinflammation. To further determine the impact of different macrophage subtypes on pain after nerve injury, we performed immunofluorescence staining to determine the distribution of IBA1‐positive and CX3CR1‐positive macrophages in the DRG. After crush, the proportions of both IBA1^+^ macrophages and CX3CR1^+^ macrophages increased (Figure 2A, C). The infiltration of IBA1^+^ macrophages was significantly greater in HFD‐fed mice (Figure 2B). Similarly, compared to that in the crush group, the proportion of CX3CR1^+^ macrophages also noticeably increased in the HFD group (Figure 2D).

**FIGURE 3:**
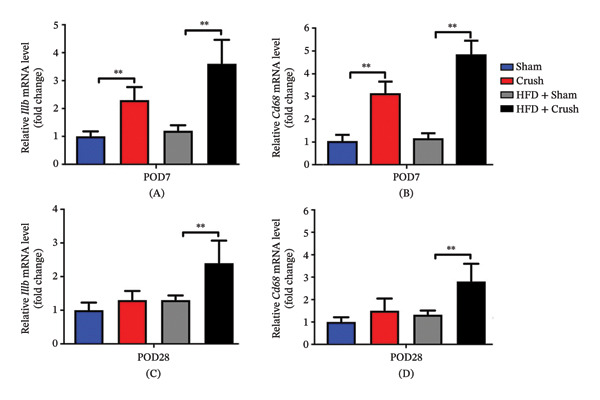
High‐fat diet exacerbates and prolongs the expression of proinflammatory markers in the DRG induced by crush injury. (A, B) PCR analysis of *Il1b* (A) and *Cd68* (B) mRNA expression in the DRG on POD 7. Crush injury upregulated these inflammatory markers compared to the sham group. This elevation was markedly amplified by HFD feeding. (C, D) mRNA expression levels of *Il1b* (C) and *Cd68* (D) in the DRG on POD 28. Although expression levels declined compared to POD 7, the HFD‐crush group maintained significantly higher levels of proinflammatory factors compared to the crush group. Two‐way ANOVA, ^∗∗^ *p* < 0.01 vs. sham. *n* = 8 per group.

To elucidate the specific mechanisms of macrophage infiltration in the development of pain, we used qPCR to quantitatively analyze the expression of cytokines secreted by activated macrophages on POD7 and POD28. On POD7, for *Il1b* and *Cd68*, the crush procedure significantly increased their mRNA expression, and HFD feeding further upregulated the expression of *Il1b* and *Cd68* (Figure 3A‐B). By POD28, the mRNA levels of the two factors were downregulated compared to those at POD7. However, the HFD group still maintained relatively higher levels of proinflammatory factors than did the other groups (Figure 3C‐D). This finding is consistent with earlier findings regarding the development of pain, where the HFD group exhibited prolonged pain duration and slower recovery.

### 3.3. MaR1 Alleviated Nerve Injury–Induced Chronic Pain and Decreased Nerve Injury–Induced GAP43 Expression in HFD‐Fed Mice

**FIGURE 4:**
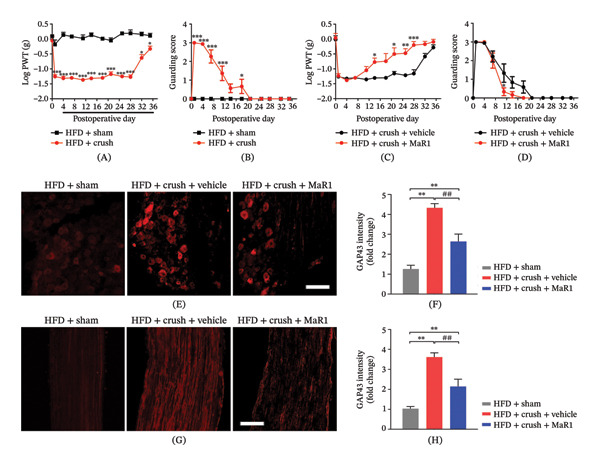
MaR1 alleviates nerve injury–induced neuropathic pain behaviors and downregulates GAP43 expression in HFD‐fed mice. (A, B) Assessment of PWT (A) and spontaneous guarding scores (B) in HFD‐fed mice following crush injury. The crush group exhibited a rapid decrease in PWT and a significant increase in guarding scores compared to the sham group, persisting until POD 20. (C, D) Intrathecal MaR1 mitigates mechanical allodynia. Compared to the vehicle‐treated group, MaR1 treatment significantly elevated PWT and reduced guarding scores. MaR1‐treated mice showed an early recovery trend initiating on POD 8. Two‐way repeated‐measures ANOVA, ^∗^ *p* < 0.05, ^∗∗^ *p* < 0.01, ^∗∗∗^ *p* < 0.001 vs. control groups. (E, F) Immunofluorescence and quantitative analysis of GAP43 expression in the DRG on POD 28. MaR1 treatment significantly reduced the aberrant upregulation of GAP43 in DRG neurons induced by crush injury under HFD conditions. (G, H) Consistent with DRG findings, MaR1 treatment significantly reduced GAP43 expression in the tibial nerve. One‐way ANOVA, ^∗∗^ *p* < 0.01 vs. HFD + sham; ^##^ *p* < 0.01 vs. HFD + crush + vehicle group. *n* = 8 per group.

As previously mentioned, MaR1 regulates nerve regeneration. To investigate the role of MaR1 in pain following nerve injury, we intrathecally injected MaR1 into HFD‐fed mice 1 day after the operation. The results showed that after crush, the paw mechanical threshold of the mice rapidly decreased compared to that of the sham group and remained at a low level until POD28 (Figure 4A), with a significant increase in guarding scores until POD20 (Figure 4B). Although the MaR1‐treated mice also exhibited significant mechanical allodynia, the mechanical allodynia of these mice was not as severe as that of the vehicle‐treated mice, and these mice showed an early recovery trend on POD8 (Figure 4C), accompanied by a decrease in guarding scores (Figure 4D). These findings indicate that MaR1 can alleviate pain and related spontaneous protective behaviors. On POD28, GAP43 staining further supported its association with axonal remodeling in the context of nerve injury and pain. MaR1 obviously reduced the expression of GAP43 in DRG neurons (Figure 4E,F) and tibial nerve fibers (Figure 4G,H) after crush injury in HFD mice.

### 3.4. MaR1 Decreased the HFD‐Induced Increase in Macrophage Infiltration in the Damaged Tibial Nerves and DRGs of Nerve Injury Model Mice

**FIGURE 5:**
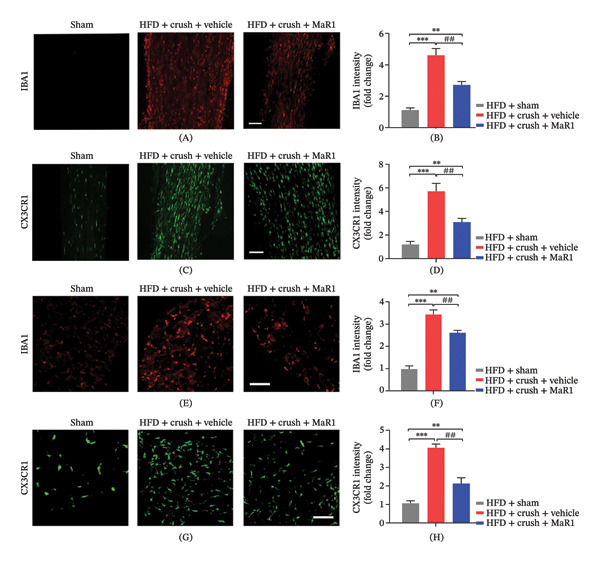
MaR1 attenuates macrophage infiltration in the injured tibial nerve and DRG of HFD‐fed mice. (A–D) Immunofluorescence images and quantitative analysis of IBA1^+^ (A, B) and CX3CR1^+^ (C, D) macrophages in the injured tibial nerve on POD 28. Compared to the vehicle‐treated group, MaR1 administration significantly reduced the infiltration density of both macrophage subtypes in HFD + crush mice. (E–H) Consistent with findings in the nerve, MaR1 markedly suppressed the recruitment of IBA1^+^ (E, F) and CX3CR1^+^ (G, H) macrophages relative to vehicle controls. One‐way ANOVA, ^∗∗∗^ *p* < 0.001, ^∗∗^ *p* < 0.01 vs. HFD + sham; ^##^ *p* < 0.01 vs. HFD + crush + vehicle group. *n* = 8 per group.

To determine the regulatory effect of MaR1 on macrophage infiltration after nerve injury, we observed the distribution of IBA1^+^ macrophages and CX3CR1^+^ macrophages in the injured tibial nerves and DRGs of HFD‐fed mice on POD28. Immunofluorescence results showed that in the tibial nerve, the infiltration of IBA1^+^ (Figure 5A,B) and CX3CR1^+^ macrophages (Figure 5C,D) markedly increased in the HFD + Crush group. After the intrathecal injection of MaR1, the proportion of IBA1^+^ macrophages (Figure 5A,B) and CX3CR1^+^ macrophages (Figure 5C,D) substantially decreased. Similar phenomena were observed in the DRGs, where both types of macrophages significantly infiltrated the DRG tissue after crush (Figure 5E,H). Following treatment with MaR1, the distribution of both IBA1^+^ (Figure 5E,F) and CX3CR1^+^ macrophages significantly decreased (Figure 5G,H).

### 3.5. MaR1 Decreased the HFD‐Induced Proinflammatory Cytokine Upregulation in the Dorsal Root Ganglion of Nerve Injury Model Mice

**FIGURE 6:**
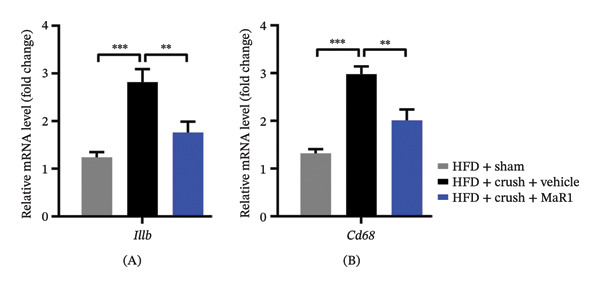
MaR1 suppresses the expression of proinflammatory markers in the DRG. (A, B) qPCR analysis of *Il1b* (A) and *Cd68* (B) mRNA expression in the DRG on POD 28. Compared to the vehicle‐treated group, MaR1 administration significantly downregulated the mRNA levels of both factors, demonstrating a robust anti‐inflammatory effect. One‐way ANOVA, ^∗∗∗^ *p* < 0.001 vs. HFD + sham; ^∗∗^ *p* < 0.01 vs. HFD + crush + vehicle group. *n* = 8 per group.

To figure out whether MaR1 has an inhibitory effect on the release of proinflammatory factors, our qPCR results indicated that, compared to the vehicle treatment, MaR1 exhibited a remarkable anti‐inflammatory effect. The expression of *Il1b* (Figure 6A) and *Cd68* (Figure 6B) was significantly suppressed after MaR1 treatment on POD28. This suggests that MaR1 may play a crucial role in modulating inflammatory responses, potentially offering therapeutic benefits in excessive inflammation and its associated pain.

## 4. Discussion

NP is a chronic and debilitating condition characterized by recurrent episodes and poor responsiveness to treatment, severely impairing patients’ quality of life. Clinically, prolonged consumption of a HFD not only predisposes individuals to metabolic disorders such as obesity and diabetes but is also strongly associated with a higher risk of NP. Previous studies have shown that HFD elevates circulating and tissue fatty acid levels, thereby triggering systemic inflammation and oxidative stress. These changes disrupt macrophages’ function, exacerbating the development and persistence of NP [18].

MaR1, an endogenous lipid mediator with immunomodulatory and tissue‐reparative properties, has emerged as a promising therapeutic candidate [13, 19, 20]. In this study, we employed a TNC model to investigate the role of MaR1 in NP under metabolic stress. We observed that, compared with the sham group, TNC significantly reduced PWTs, and the effect was further aggravated by HFD feeding, manifesting as prolonged and intensified mechanical allodynia. HFD‐fed mice also exhibited upregulated expression of GAP43, a marker of axonal growth and neural plasticity, in the DRGs and tibial nerve fibers. This was accompanied by substantial infiltration of IBA1‐positive and CX3CR1^+^ macrophages, along with increased expression of proinflammatory mediators such as IL‐1β and Cd68.

Notably, MaR1 administration produced a significant and early attenuation of pain‐related behaviors in HFD‐fed mice, with analgesic effects evident as early as POD8. From POD14 to POD28, PWT values in the HFD + crush + MaR1 group showed a clear separation from those in the HFD + crush + vehicle group, indicating a faster and more robust recovery trajectory that gradually stabilized by POD36. Furthermore, MaR1 downregulated GAP43 expression and macrophage infiltration in the DRGs and injured nerves. It also decreased the expression of inflammatory factors IL‐1β and Cd68 at POD28, thereby alleviating inflammation. Collectively, these findings indicate that MaR1 simultaneously modulates neuroinflammatory responses and aberrant nerve regeneration.

Adipose tissue has been reported to secrete MCP‐1, which facilitates the recruitment and polarization of CCR2^+^ monocytes into proinflammatory M1 macrophages. These macrophages, once infiltrated into white adipose tissue (WAT), release cytokines such as TNF‐α, IL‐1β, and IL‐6, contributing to systemic inflammation [21]. Liang et al. [22] demonstrated that HFD‐fed neuropathic mice displayed increased spinal microglial activation and M1 phenotype skewing over time, with a concomitant rise in TNF‐α levels. Ablation of microglia alleviated mechanical hypersensitivity and decreased TNF‐α mRNA expression. Similarly, Zhang et al. [23] found elevated levels of Cd68^+^ macrophages and proinflammatory cytokines in the serum and lumbar DRGs of HFD‐fed mice, alongside a reduction in anti‐inflammatory IL‐10. In diabetic NP models, macrophage depletion alleviated mechanical allodynia and reduced levels of IL‐1β, CCL3, and CCL4 [24]. HFD feeding also exacerbated muscle and systemic inflammation in acid‐induced hyperalgesia models, with marked TNF‐α upregulation across multiple tissues including the DRG and spinal cord [25].

These data collectively suggest that HFD potentiates neuroimmune crosstalk, potentially through parasympathetic nervous system mechanisms. The vagus nerve, activated by adipose‐derived cytokines, can modulate inflammation via *α*7 nicotinic acetylcholine receptors (*α*7nAChR) on macrophages, inhibiting TNF‐α production [26]. Moreover, neuropeptides like CGRP and substance P from WAT may amplify immune cell influx and polarization, heightening sensory nerve sensitization and promoting NP [21].

Our study further demonstrated that HFD significantly increased GAP43 expression following injury, whereas MaR1 treatment markedly reduced GAP43 levels in both the DRG and tibial nerve, concurrently alleviating pain behavior. Existing literature indicates that an HFD induces lipotoxicity, oxidative stress, and sustained elevation of inflammatory factors. These changes significantly alter the reparative microenvironment following nerve injury and disrupt normal axonal regeneration programs [27, 28]. In this context, the significant upregulation of GAP43 postinjury may not represent effective nerve repair; rather, it likely reflects an amplified response of maladaptive regeneration or structural plasticity occurring under adverse metabolic and inflammatory conditions.

Recent studies have shown that following peripheral nerve injury, GAP43 and its phosphorylated form (pGAP43) remain persistently elevated in the DRG and their central terminals. This elevation is closely associated with the morphological remodeling of injured C‐fiber terminals, an aberrant process believed to contribute to the maintenance of chronic pain [29]. Mechanistically, inflammation‐related signaling pathways, particularly the JNK pathway [29], mediate the persistent phosphorylation of GAP43. This drives abnormal morphological changes in nociceptive nerve terminals, including terminal swelling, synaptic remodeling, and the formation of neuroma‐like structures [30]. Conversely, inhibiting JNK activity or reducing pGAP43 expression has been shown to alleviate pain behaviors [29], suggesting that GAP43‐associated neural plasticity possesses distinct pathological significance rather than serving merely as a marker of regeneration. Moreover, evidence indicates that aberrant activation of axonal regeneration or structural plasticity, such as excessive synaptic reconstruction or disorganized nerve terminal growth, can amplify nociceptive input and sustain central sensitization, thereby promoting the development and persistence of chronic pain [30]. Together, these findings support the view that the elevated GAP43 expression observed under conditions of inflammation or metabolic stress (such as HFD) reflects an imbalanced and maladaptive regenerative or plastic response.

Although direct evidence of MaR1 modulating JNK in the context of pain is limited, studies in other inflammation and injury models have found that MaR1 significantly inhibits JNK phosphorylation and overall MAPK signaling activity. This supports the hypothesis that MaR1 may influence the pathological regulation of GAP43 by suppressing JNK‐associated signaling [31]. Additionally, as a specialized proresolving lipid mediator derived from macrophage metabolism of DHA, MaR1 plays a crucial role in dampening inflammatory cascades and promoting repair [25]. Intrathecal MaR1 has been shown to suppress carrageenan‐induced mechanical and thermal hyperalgesia, reduce CGRP^+^ fiber‐associated neutrophil recruitment, and inhibit glial activation and inflammatory signaling in the spinal cord. Specifically, MaR1 downregulates NF‐κB, IL‐1β, and TNF‐α and suppresses DRG neuron activation by reducing Nav1.8 and TRPV1 expression [19]. Additional evidence suggests that MaR1 inhibits NF‐κB translocation, limits NLRP3 inflammasome activation, and shifts macrophage polarization toward the M2 phenotype, marked by increased MRC1, IL‐10, and TGF‐β expression [17, 32–34].

Although pharmacological inhibitors or genetic knockout models were not used to directly verify the downstream targets of MaR1 in the present study, our findings are highly consistent with previous reports. In particular, MaR1 treatment was associated with reduced macrophage infiltration and activation in the DRGs and injured nerves, along with decreased expression of IL‐1β and CD68 (a marker of M1 polarization), suggesting an attenuation of the HFD‐induced inflammatory microenvironment. Inflammatory mediators are known to activate the PI3K/Akt signaling pathway [35], a core cascade that governs axonal regeneration, growth cone formation, and neuronal survival [36]. Given this connection, we speculate that MaR1 exerts its analgesic effects by suppressing inflammation, thereby indirectly downregulating GAP43 expression and preventing maladaptive regenerative signaling.

Our study underscores the pivotal role of inflammation in HFD‐associated NP and highlights the potent therapeutic potential of MaR1. However, we acknowledge a limitation in discerning the precise cellular targets of MaR1‐specifically, whether it acts directly on neurons or indirectly via the modulation of macrophages or microglia. In our gene expression analysis, qPCR was performed on whole DRG tissue lysates. Consequently, the observed downregulation of proinflammatory markers (e.g., *Cd68*, *Il1b*) reflects a suppression of inflammation at the tissue level, rather than a strictly cell‐specific effect. We cannot fully rule out the possibility that MaR1 may also exert direct protective effects on neurons or other glial cells. Nevertheless, our immunofluorescence data (Figure 5) provide critical spatial resolution, clearly demonstrating a significant reduction in the infiltration and density of CD68‐positive macrophages in the MaR1‐treated group. Taken together, while the potential contribution of other cell types remains to be fully elucidated, the parallel downregulation of macrophage markers and cytokine levels strongly suggests that inhibiting macrophage recruitment and activation is a key component of MaR1‐mediated anti‐inflammatory action. Future studies utilizing in vitro coculture systems or conditional knockout models will be instrumental in further dissecting the direct versus indirect mechanisms of MaR1 on specific cellular populations. In summary, our findings indicate that HFD may exacerbate neuroinflammation and disrupt regenerative balance, thereby contributing to the aggravation of NP. MaR1 exerts potent therapeutic effects by modulating macrophage infiltration, attenuating inflammatory responses, and influencing nerve regeneration. Although this study did not extend follow‐up to a longer duration, limiting a comprehensive assessment of long‐term efficacy, the current results confirm that MaR1 significantly alleviates pain and promotes early recovery during the critical transition window to chronic pain. From a clinical perspective, this study not only elucidates the mechanisms underlying HFD‐exacerbated pain but also expands the therapeutic potential of MaR1 in metabolic neuropathies, such as diabetic neuropathy and obesity‐related neuropathy. The HFD‐induced injury microenvironment shares critical pathological features with metabolic neuropathies, including chronic low‐grade inflammation, dysregulated neuroimmune crosstalk, and maladaptive neural plasticity [37, 38]. Notably, our findings suggest that MaR1 may possess a unique dual therapeutic potential: It appears to simultaneously resolve metabolic inflammation (e.g., downregulating IL‐1β and CD68) and attenuate GAP43‐mediated maladaptive neuroplasticity. This distinct mode of action implies a therapeutic strategy that differs from sole glycemic control or symptomatic analgesics. Therefore, MaR1 holds promise as a novel endogenous disease‐modifying strategy, offering new intervention insights for addressing the inflammation resolution deficit and regenerative dysregulation in neuropathies under metabolic stress.

**Nomenclature**
NP: neuropathic pain
DH: dorsal horn
DRG: dorsal root ganglion
CGRP: calcitonin gene–related peptide
WAT: white adipose tissue
ASC: apoptosis‐associated speck‐like protein containing a CARD
*α*7nAChR: *α*7 nicotinic acetylcholine receptor
CSF1: colony‐stimulating factor 1

## Author Contributions

Guokai Liu, Li Zhang, and Fan Liu designed the experimental protocol, drafted the manuscript, and revised the article. Ran Hu, Kexin Qin, Xue Wang, Chen Wu, and Zichen Liu conducted the experiments and wrote the initial draft of the manuscript. Kexin Qin, Xue Wang, Xiangqi Shao, Jianru Sun, Yang Liu, Saisong Xiao, and Huilong Ren analyzed the experimental data and made the result figures. All authors made significant contributions and are responsible for the experimental results.

## Funding

This study was supported by the National Natural Science Foundation of China (Grant No. 82271260), the Fundamental Research Funds for the Central Universities (Grant No. 2024‐JYB‐JBZD‐024), the CZ015‐High Level Traditional Chinese Medicine Hospital SM Project (Grant No. DZMG‐LJRC0006), Capital Medical University Research Foundation (Grant No. PYZ22065), and the Beijing Natural Science Foundation (Grant No. 7232103).

## Ethics Statement

All animal procedures performed in this study were reviewed and approved by the Institutional Animal Care and Use Committee of Beijing Friendship Hospital, Capital Medical University (20–1004), and were conducted in accordance with the guidelines of the International Association for the Study of Pain.

## Conflicts of Interest

The authors declare no conflicts of interest.

## Data Availability

The data that support the findings of this study are available from the corresponding author upon reasonable request.
